# Bilateral Iridochorioretinal Coloboma Managed with Low Vision Rehabilitation: A Case Report

**DOI:** 10.31729/jnma.8023

**Published:** 2023-02-28

**Authors:** Basanta Singh, Rinkal Suwal, Rashmi Shrestha, Sikshya Adhikari, Sudip Karki, Deepak Khadka

**Affiliations:** 1 Department of Ophthalmology, B.P. Eye Foundation, Hospital for Children, Eye, ENT, and Rehabilitation Services, Madhyapur Thimi, Bhaktapur, Nepal

**Keywords:** *case reports*, *coloboma*, *ocular*, *rehabilitation*, *training*

## Abstract

Ocular coloboma is a rare congenital disability. If involving the macula, it affects the patient's vision and subsequently affects childhood development and quality of life in the future. Appropriate low vision aid and timely rehabilitation can provide the best possible quality of life for visually impaired children. We report a 9-year-old boy who presented with a diminution of vision in both eyes, and who was just enrolled in pre-school. He was diagnosed with bilateral iridochorioretinal coloboma associated with nystagmus and unilateral cataract. After all the necessary evaluation, a telescope was prescribed for distance and a dome magnifier for near. Furthermore, a peaked cap and photo grey lens were given for outdoor activities. This case highlights the importance of low vision intervention in a visually impaired child. Appropriate low vision aid and rehabilitation can improve patients' lifestyle and academic performance who are diagnosed with iridochorioretinal coloboma.

## INTRODUCTION

Ocular coloboma is a rare congenital abnormality because of defective choroidal fissure closure, usually occurring in the 6^th^ and 7^th^ weeks of fetal life.^1^ It may involve the uveal tract, retina, and optic nerve. If coloboma involves the macula, vision is affected. A bilateral decrease of vision can affect childhood development.^2^ Low vision rehabilitation focuses on people's lives with impaired vision by improving functional ability, psychosocial condition, and quality of life.^3^ Low Vision Aids (LVAs) include optical, non-optical as well as electronic devices. These devices are highly effective in enhancing visual acuity (VA) and quality of life in children with impaired vision.^4^

## CASE REPORT

A 9-year-old boy presented to the tertiary care Hospital with the chief complaint of diminution of vision in the right eye for 2 months and the left eye by birth. He was accompanied by his father, who also complained of rapid eye movement since birth. Birth history was normal full-term with home delivery in the absence of consanguinity. The prenatal history was unremarkable. His parents were healthy and he had two unaffected siblings. Although he was 9 years old, he was just enrolled in preschool. The patient was referred to a low-vision clinic after he was diagnosed with bilateral iridochorioretinal coloboma.

**Figure 1:**
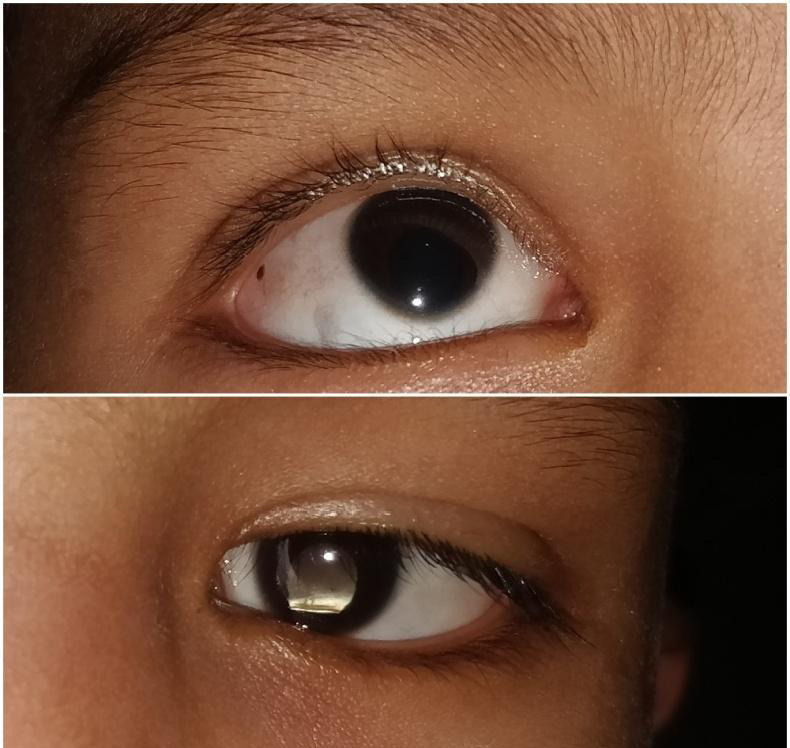
Iris coloboma in both eyes.

The patient had no previous history of low vision examination. He informed having difficulty recognizing faces, watching television, and copying from the board in pre-school. There was trouble reading the small print on near task, and he was using his mobile phone very closely. His father also enlightened mobility problems in unfamiliar places and bumping into objects, especially during the night. He had a glaring problem in bright light, and there was no history of difficulty in color perception. His best-corrected visual acuity was 3/60 (1.3 logMAR) in the right eye and 6/600 (2.0 logMAR) in the left eye on ocular examination. Near vision was 2.0 M (N16) at 25 cm in both eyes when measured from the Landolt ring chart. There was no improvement in vision with refraction. Color vision was within the normal limit. Contrast sensitivity was 2.5% in the right eye and 25% in the left eye measured at 40 cm with low contrast flip chart (LEA symbol). General appearance showed abnormal head posture with face turned towards right and head tilted towards left. There was a central corneal reflex with the Hirschberg test. Extraocular motility was full, free, and painless. Manifest latent jerky horizontal nystagmus was noted in both eyes. The horizontal visible iris diameter was 9 mm in both eyes, and the vertical visible iris diameter was 10 mm in the right eye and 9 mm in the left eye. Axial length was 21.11±0.02 mm and 21.43±0.03 mm in the right eye and left eye. Lens thickness was 4.01±0.04 mm in the right eye and 4.34±00.27 mm in the left eye. Anterior segment examination showed iris coloboma in the inferonasal quadrant in both eyes (Figure 1).

**Figure 2:**
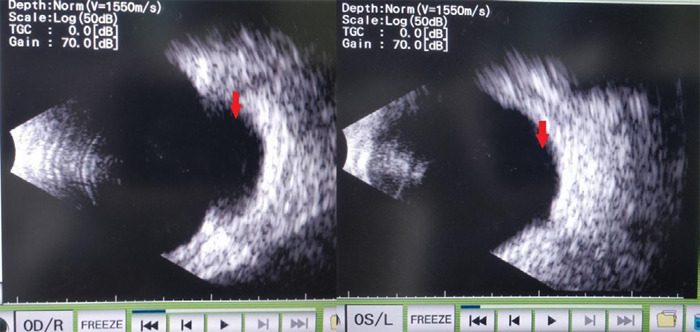
B scan report of right and left eye showing chorioretinal coloboma (pointed by red arrow) in inferonasal quadrant.

In the left eye, the visual axis was clear; however, an inferior lamellar cataract was noted. In both regards, fundus examination revealed choroidal coloboma involving the disc and part of the macula. Furthermore, B-scan was done (Figure 2). On the contrary, Fundus's photo could not be taken because of nystagmus.

**Figure 3:**
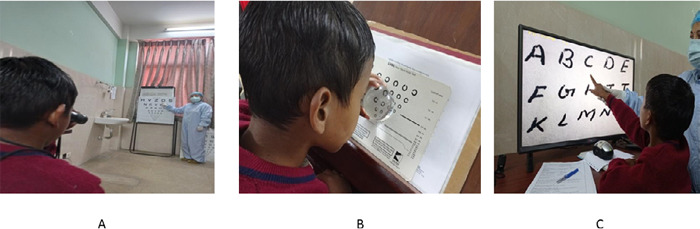
A) Training for the use of a monocular telescope, B) Dome magnifier training, C) CCTV training.

A low vision device trial was done in the right eye for distance and near (Figure 3A). A 3X handheld monocular telescope improved VA up to 0.6 logMAR (6/24). In addition, the 4X monocular telescope improved VA up to 0.5 logMAR (6/18). Further, the 2X dome magnifier improved near VA to 1M (N8) at 25cm which is sufficient for continuous reading of a 2M print-size children's book (Figure 3B). In the trial of Absorptive lenses, he was more comfortable with grey filters. In addition, the patient was also trained with Closed Circuit Television (CCTV) (Figure 3C).

However, the patient and attender were from a remote place in Nepal and did not find it practical due to its less portable nature. Therefore, we planned to dispense 3X monocular handheld telescope and 2X Dome Magnifier after proper training. The patient's attender was explained about the nature and progression of the disease. A photochromatic lens (photo grey) and a peaked cap were prescribed for outdoor activities. Approach magnification was explained while reading, writing, and recognizing faces. Reading stand was advised for comfortable body posture. Educational counseling was done.

We advised guardians to visit the hospital for a month for Low Vision Rehabilitation. For the first couple of days, the child was allowed to be familiar with the optical instrument. Although he was reluctant to use the telescope at first, he was gradually getting fond of it. However, he was comfortable using a dome magnifier. Initially, he was asked to align the eye and telescope with the target. Later, an explanation about focusing the telescope on clearing the letters on the board and copying those letters in a notebook was given. In the second week, we mainly concentrated on the scanning and tracing part of the training. He was asked to scan a whiteboard to find a particular letter or trace a line to locate a target. He also started copying words from the board to his notebook. In the last two weeks, we focused on tracking a moving target like tracking a moving person. Simultaneously, we also explained how to use a dome magnifier by asking the patient to search and find a particular alphabet on a given page. After one month of training, the child was comfortable using the telescope and magnifier. His speed of copying the words and sentences from the whiteboard to his notebook gradually increased over time. Finally, a monocular telescope and dome magnifier were dispensed for everyday use. A letter to the school teacher was given for school sitting modification and the use of a telescope while blackboard viewing. In addition, we were in touch with the teacher throughout this period regarding any improvement and child performance.

## DISCUSSION

Ocular coloboma has annual incidence of 2.4 per 100000 residents <19 years old and prevalence of 2077 live births.^5^ Older paternity age, history of prematurity, low birth weight, and alcohol consumption in pregnancy are some of the risk factors in coloboma development.^6^ Ocular coloboma can be translated as an autosomal dominant, autosomal recessive, sporadic, or X-linked trait.^1^ However, there were no known risk factors or genetic history in our case.

Ocular coloboma can have other ocular and systemic associations as well. Cataract, strabismus, microphthalmia, anophthalmia, nystagmus, anisometropia, hypoplasia of the optic nerve, and retinal detachment are some ocular associations. In addition, systemic involvement may include abnormal development, heart anomalies, ear anomalies, seizures, skeletal anomalies, and urogenital anomalies.^5^ Here, this case had nystagmus and microcornea with no other systemic association. Retinoscopic reflex showed a clear glow in the visual axis. In the left eye, a lamellar cataract was present but insignificant and not in the visual axis; therefore, cataract surgery was not advised. In the case of nystagmus, if the null point is eccentric, the patient takes up a compensatory head posture to improve vision.^7^ Here in our case, the patient had a compensatory face turn towards the right and head tilt towards the left.

Low vision is a permanent visual impairment that affects VA, visual field, or contrast sensitivity that cannot be corrected by refraction, medicine, or surgery.^8^ Patients with low vision may have difficulty with daily living skills, leading to lower quality of life and maybe even loss of independence.^9^ In our case, the patient was 9 years old and this was his first low vision assessment. He was just enrolled in preschool and was having difficulty reading from the blackboard. Earlier intervention and rehabilitation could have made him capable of performing basic classroom activities. Furthermore, a study done in 2015 reported a 4-year-old boy who benefitted from LVA.^4^ This reflects the positive role of LVAs in enhancing visual acuity at a very young age and highlights the importance of early referral. An earlier referral provides adequate time for patients to develop a relationship with the low vision team and LVAs and is pivotal in the case of childhood visual impairment.^10^ Visual impairments can hamper with the academic environment. Early rehabilitation may boost children's comfort with LVAs improving their functional vision and allowing them to perform better in school.^11^ Besides, distance VA and near reading performance can be enhanced by using optical LVAs in children with low vision due to bilateral chorioretinal coloboma.^12^ The use of a telescope and magnifier significantly improved reading performance in our patient as well.

Low vision services are more than just providing low vision aids. Proper training regarding the use of optical and non-optical aids is also required for longterm success. A poor low vision service is worse than no low vision service because it affects motivation.^13^ A thorough training about the proper use of low vision aids can decrease the failure rate from 22% to 3%.^14^ Additionally, modern Low vision service focuses more on restoring quality of life, so we have trained our patients for one complete month to ensure sustainability.^15^ Parents and teachers were asked to help him in the process of adaptation. The use of low vision aid has improved our patient's academic performance and thus highlights its effectiveness in cases of ocular coloboma.

To conclude, low vision rehabilitation is more focused on improving the patient's quality of life. It can take the central stage in the services we provide for patients with iridochorioretinal coloboma if implemented properly. Early low-vision intervention in congenital cases and proper low-vision rehabilitation can ensure independence in the future. Awareness among the general public, health workers, and the government becomes pivotal for successfully implementing Low vision services.
